# Shedding Light on People Action Recognition in Social Robotics by Means of Common Spatial Patterns

**DOI:** 10.3390/s20082436

**Published:** 2020-04-24

**Authors:** Itsaso Rodríguez-Moreno, José María Martínez-Otzeta, Izaro Goienetxea, Igor Rodriguez-Rodriguez, Basilio Sierra

**Affiliations:** Department of Computer Science and Artificial Intelligence, University of the Basque Country, Manuel Lardizabal 1, 20018 Donostia-San Sebastián, Spain; josemaria.martinezo@ehu.eus (J.M.M.-O.); izaro.goienetxea@ehu.eus (I.G.); igor.rodriguez@ehu.eus (I.R.-R.); b.sierra@ehu.eus (B.S.)

**Keywords:** action recognition, social robotics, common spatial patterns

## Abstract

Action recognition in robotics is a research field that has gained momentum in recent years. In this work, a video activity recognition method is presented, which has the ultimate goal of endowing a robot with action recognition capabilities for a more natural social interaction. The application of Common Spatial Patterns (CSP), a signal processing approach widely used in electroencephalography (EEG), is presented in a novel manner to be used in activity recognition in videos taken by a humanoid robot. A sequence of skeleton data is considered as a multidimensional signal and filtered according to the CSP algorithm. Then, characteristics extracted from these filtered data are used as features for a classifier. A database with 46 individuals performing six different actions has been created to test the proposed method. The CSP-based method along with a Linear Discriminant Analysis (LDA) classifier has been compared to a Long Short-Term Memory (LSTM) neural network, showing that the former obtains similar or better results than the latter, while being simpler.

## 1. Introduction

Social robotics aims at providing robots with artificial social intelligence to improve human–machine interaction and to introduce them in complex human contexts [1]. An effective social interaction between humans and robots requires these robots to understand and adapt to the human behaviour. Using visual perception for human activity recognition will aid the robot to provide better responses and thus enhance its social capabilities. The robot will be able to understand when the user wants to engage with it by recognising the action she/he performs.

Human activity recognition in videos is a task which consists in recognising certain actions from a series of observations. This field of research has received great attention since 1980 due to the amount of applications for which it is useful, such as health sciences, human-computer interaction, surveillance or sociology [2]. For example, in the field of surveillance [3], the automatic detection of suspicious actions allows an alert to be sent and some measures to be taken to deal with the danger. Another example is the use of action recognition for rehabilitation, which involves recognising the action the patients perform and being able to determine if they are performing it correctly or incorrectly. The principal field where this task is studied is in computer vision, based on videos. The visual features of a video provide basic information of the events or actions that occur.

Understanding what is happening in a video is really challenging, and different features can be taken into account when analysing a video sequence. For example, Video Motion Detection is a constrained approach which consists in detecting the movement in a static background. On the other hand, Video Tracking focuses on associating objects in consecutive frames, which can be difficult if the objects are moving fast in relation to the frames per second rate. Moreover, if the object in the scene must be recognised (already a challenging task), an additional complexity is added to the problem.

In the last few years many attempts to solve these problems have been made using different techniques such as Optical Flow, Hidden Markov Models (HMM) or, more recently, deep learning [4,5]. For example, the authors of [6,7] use Histograms of Optical Flow to perform recognition. However, in [8,9] the authors use the depth information obtained by depth cameras (Microsoft Kinect or Inter Realsense), due to the fact that depth images provide additional useful information about movement. The work of [10] must also be mentioned, as it is a reference for methods that use deep learning for this task. The authors propose a two-stream architecture incorporating spatial and temporal networks, which has been used in many subsequent methods.

Considering the computational cost and the complexity that come from the need of combining temporal and spatial information, the video classification problem progresses slowly when compared with image classification.

In this paper, a new approach for video action recognition is presented. The Common Spatial Pattern algorithm is used, a method normally applied in Brain Computer Interface (BCI) for EEG systems [11]. Videos are recorded and processed with OpenPose [12] software in order to obtain a sequence of skeleton data. This skeleton data corresponds to the position of the joints of the person performing the action of the video. A sequence of skeleton data is extracted from the video, and this data can be treated as a multidimensional signal. It is then filtered according to the Common Spatial Patterns (CSP) algorithm and characteristics extracted from these filtered data are used as features for a classifier. Linear Discriminant Analysis and Random Forest (RF) classifiers have been tested to build the models from the features extracted in the previous step. Variance, maximum, minimum and interquartile range (IQR) of the filtered signals have been taken as features to feed the aforementioned classifiers. The spatial filter generated by CSP is employed as a dimensionality reduction approach and can also be interpreted in EGG data analysis as a technique that sheds light on the relationships between the filtered signals, in a similar manner to Principal Component Analysis [13] (PCA), from which it is derived. While no direct visual interpretation is possible when applied to skeleton data, this dimensionality reduction technique allows for extracting the signal components which maximally discriminate between classes.

**Figure 1:**
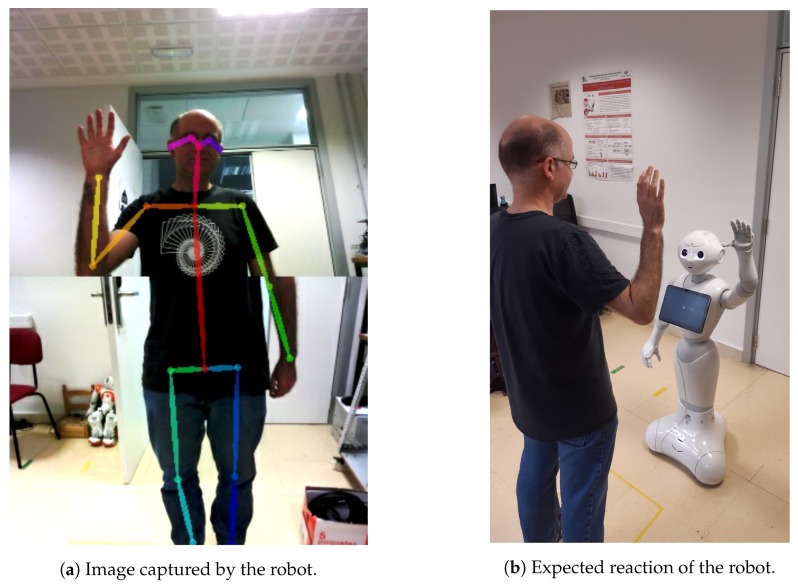
Interaction example.

In Figure 1 an interaction example of a person with the robot is displayed. On the left, the skeleton superposed over the actual person that is interacting with the robot is shown. The skeleton contains the (X,Y) position of 25-keypoints, which include body, head and feet information. On the right, another point of view can be seen, with the expected response of the robot. A more detailed explanation about the employed human pose estimation system and the skeleton definition is provided in Section 4.1.

**Figure 2:**
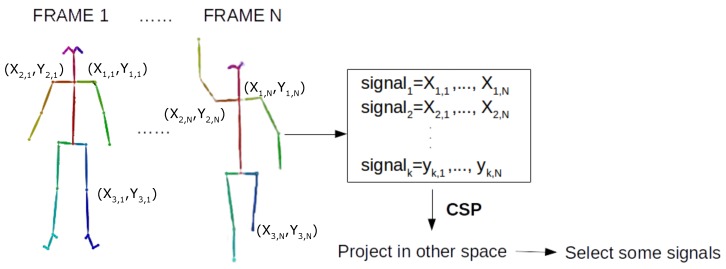
Proposed approach.

To apply CSP, as a first step, the skeleton of the person appearing in each frame is extracted using OpenPose, and the (X,Y) position of each of the 25 joints that OpenPose detects are used as input data to the CSP. Therefore, in the presented method, input videos are represented as frame sequences and the temporal sequence of each skeleton joint is treated as an input signal (channel) to the CSP. In Figure 2, the following data acquisition process is shown.

In order to validate the proposed CPS-based approach, an experiment is performed where it is compared with Long Short-Term Memory [14] neural networks, yielding better results.

The rest of the paper is organised as follows. First, in Section 2 some related works are mentioned in order to introduce the topic. In Section 3 a theoretical framework is presented to explain the proposed algorithm in detail.

In Section 4 the used dataset and related skeleton capture system, as well as the experimentation carried out, are explained thoroughly, and the obtained results are shown, including a comparison between the presented approach and a Keras [15] implementation of a LSTM network. A brief introduction to LSTMs is also presented in this section. The paper concludes with the Section 5, where the conclusions from the presented work are presented and some future work is pointed out.

## 2. Related Work

As activity recognition has been an active research area lately, many different strategies have been developed to deal with this problem. There are several ways to extract visual features, both static image features and temporal visual features, and subsequently use them to perform the recognition. Temporal visual features are a combination of static image features and time information, so through these features temporal video information is achieved.

In [16] the authors use a temporal template as the basis of their representation, continuing with their work presented in [17]. This temporal template consists of a static vector-image where the value of the vector at each point represents a function of the motion properties at the corresponding spatial location in an image sequence. The authors of [18] demonstrate that local measurements in terms of spatio-temporal interest points (local features) can be used to recognise complex motion patterns. In [19] the authors present a hybrid hierarchical model, where video sequences are represented as collections of spatial and spatio-temporal features. These features are obtained by extracting both static and dynamic interest points, and the model is able to combine static and motion image features, as well as to perform categorisation of human actions in a frame-by-frame basis. Laptev et al. [20] contribute to the recognition of realistic videos and use movie scripts for automatic annotation of human actions in videos. Due to the promising performance that they achieve in image classification [21,22,23,24], they employ spatio-temporal features and spatio-temporal pyramids, extending the spatial pyramids presented in [22].

Many other methods make use of the optical flow to solve this issue. Optical flow is the motion of objects between consecutive frames, caused by the relative movement between an observer and a scene. Therefore, optical flow methods try to calculate the motion between two image frames which are taken at times *t* and t+Δt at every position, assuming that the intensity of objects does not change during the movement. The authors of [6] use Histograms of Oriented Gradients (HOG) for human pose representations and time series of Histogram of Oriented Optical Flow (HOOF) to characterise human motion. In [7], the authors also use HOOF features for frame representation, which are independent to the scale of the moving person and to the direction of motion. There are many approaches which are based on histograms [25,26,27]. The authors of [28] introduce a motion descriptor based on the direction of optical flow, using the Lucas–Kanade algorithm [29] to compute it. In [30], the authors defend that to deal with the video-based action recognition problem temporally represented video information is needed. In their work, optical flow vectors are grouped according to their angular features and then summed and integrated with a new velocity concept.

It should also be mentioned that the interest of using depth data captured by depth cameras for the action recognition problem has grown, due to the advances in imaging technology to capture depth information in real time, and there are many approaches which use this extra information to make the recognition [8,9,31,32].

Some works focus on using skeleton data to perform activity recognition. In [33], the authors present a representation for action recognition, for which they use a human pose estimator and extract heatmaps for the human joints in each frame. Ren et al. [34] proposed a method for encoding geometric relational features into colour texture images, where temporal variations of different features are converted into the colour variations of their corresponding images. They use a multistream CNN model to classify the images.

As a result of the great performance that deep learning methods have achieved in image classification, these techniques have also been applied to video-based activity recognition. Taking these two publications [10,35] as a starting point, deep learning has continued to be used for activity recognition, mainly with Convolutional Neural Networks (CNN) and LSTMs. Wang et al. in their work [36] presented a very deep two-stream CNN in order to improve the results of recent architectures, getting closer to image domain deep models. In [37], trajectory-pooled deep-convolutional descriptor (TDD) is introduced, where the authors first train two-stream CNNs and then use them as feature extractors to achieve convolutional spatial and temporal feature maps from the learned networks. In the work of Feichtenhofer et al. [38], authors show that it is important to associate spatial feature maps of a particular area to temporal feature maps for that corresponding region. Authors of [39] proposed an action recognition method by processing the video data using Convolutional Neural Networks and deep bidirectional LSTM (DB-LSTM) networks. The use of deep learning for video recognition is still a work in progress, and even though the obtained results are not as good as those obtained in image recognition, better results are being achieved.

## 3. CSP-Based Approach

The core motivation of the presented method is to treat temporal sequences of skeleton joints as signals to be later processed with the CSP algorithm. In this section the CSP algorithm and the proposed approach, which makes use of that algorithm, are introduced.

### 3.1. CSP

In the last few years, the Common Spatial Pattern algorithm (first mentioned in [40] as Fukunaga-Koontz Transform) has been widely used in Brain Computer Interface (BCI) applications for electroencephalography (EEG) systems [41,42,43]. It is a mathematical technique used in signal processing and it consists in finding an optimum spatial filter which reduces the dimensionality of the original signals. CSP was presented as an extension of Principal Component Analysis. Considering just two different classes, a CSP filter maximises the variance of filtered signals of EEG of one of the targets while it minimises the variance for the other, in this way maximising the difference of the variances between the classes.

The feature extraction is organised in the following way:

Let $X_{1}$ and $X_{2}$ denote two sets of *n* signals where a signal is a sequence of values read from a sensor. First the covariance matrices are computed as in (1).

(1) $$R_{1}=\frac{X_{1}X_{1}^{T}}{trace(X_{1}X_{1}^{T})};\;R_{2}=\frac{X_{2}X_{2}^{T}}{trace(X_{2}X_{2}^{T})}$$

Then, the eigen decomposition of the composite spatial covariance matrix is computed as in (2), where λ is the diagonal matrix of eigenvalues and *U* is the normalised eigenvectors matrix. To scale the principal components, the whitening transformation is used (3), obtaining an identity matrix as covariance and variance 1 for each variable.

(2) $$R_{1}+R_{2}=U\lambda U^{T}$$

(3) $$P=\sqrt{\lambda^{-1}}U^{T}$$

$R_{1}$ and $R_{2}$ covariance matrices are transformed using *P* (4). After that, taking into account that the sum of two corresponding eigen values is 1 ($\psi_{1}+\psi_{2}=I$), the eigen decomposition is computed in order to find their common eigenvectors (5).

(4) $$S_{1}=PR_{1}P^{T};\;S_{2}=PR_{2}P^{T}$$

(5) $$S_{1}=V\psi_{1}V^{T};\;S_{2}=V\psi_{2}V^{T}$$

The CSP filters are obtained as in (6), which maximises the separation between both classes. Using *W* as a projection matrix (just the first *q* and the last *q* vectors), each trial can be projected, obtaining a filtered signal matrix as in (7).

(6) $$W=P^{T}V$$

(7) $$Z=W^{T}X$$

The feature vector to be created for classification purposes is shown in (8), where $var_{p}\left(Z_{i}\right)$ is the variance of the row *p* of the *i*-th trial of *Z*. The feature vector value for the *p*-th component of the *i*-th trial is the logarithm of the normalised variance. The feature vector has 2q dimensionality, where *q* indicates how many vectors of the spatial filter are used in the projection. Exactly, *q* first and *q* last vectors are used, which yield the smallest variance for one class and simultaneously, the largest variance for the other class.

(8) $$f_{p}^{i}=log\left(\frac{var_{p}\left(Z_{i}\right)}{\sum_{p=1}^{2q}var_{p}\left(Z_{i}\right)}\right)$$

The purpose of this algorithm is to filter the data so their variance could be used to discriminate two populations, that is, to separate the signals belonging to two different classes. This algorithm can be useful in action recognition, where actions belonging to different classes have to be separated. From each video a group of signals is extracted (in the proposed approach, the coordinates of the joints’ positions), and then, the CSP algorithm filters the signals in a way that maximum variance difference is obtained for two different classes. Features from the filtered data obtained by CSP are therefore used as input to a classification algorithm to discriminate instances that belong to different classes.

### 3.2. Proposed Approach

Even though the CSP algorithm has been used mainly with EEG problems, in this paper a new application is presented; the use of CSP filters for feature extraction in the human action recognition task. In the presented method, each video represents a trial and each skeleton joint is treated as an EEG channel, so the videos are taken as time series where the joints of the extracted skeletons are the channels which change over time.

In Brain–Computer Interface, some electrodes are placed along the scalp and they are used to record the electrical activity of the brain. Therefore, the signals are obtained from the electrodes and then the CSP is applied using the electroencephalography signals.

However, in the proposed approach, the signals used to feed the CSP are obtained in another way. The full process can be seen in Figure 2, where the signals are composed with keypoints of the skeleton of the actor who is performing the action to recognise. Each trial is a video where the signals are the values of the skeleton position over time. Once the skeletons are processed and, hence, the signals are formed, the CSP is computed in order to separate the classes according to their variance.

The main focus of the experimentation is the use of the variance of the signals after applying the Common Spatial Pattern algorithm as input to the classification algorithms. However, in addition to the variance, much more information can be extracted from these transformed signals, which may be useful when performing the classification. Hence, some experiments are performed with just the information of the variances and other experiments also with information about the maximum, minimum and the interquartile range (IQR=Q3−Q1) of the signal. Once the features are extracted from the transformed signals, Linear Discriminant Analysis and Random Forest classifiers are used to perform the classification. The Linear Discriminant Analysis [44] tries to separate the different classes by finding a linear combination of features which describe each of the targets. Random Forest [45] is a Bagging (Bootstrap Aggregating) multiclassifier composed of decision trees.

## 4. Experimental Results

### 4.1. Robotic Platform and Human Pose Estimation

**Figure 3:**
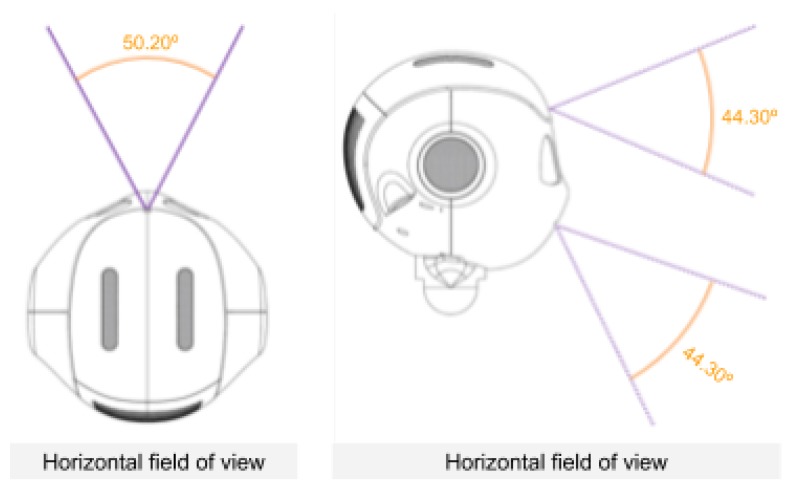
Pepper’s RGB cameras position and orientation.

The robotic platform employed in the performed experiments is a Pepper robot developed by Softbank Robotics (https://www.softbankrobotics.com/emea/en/pepper). Pepper is a human-like torso that is fitted onto a holonomous wheeled platform. It is equipped with full-colour RGB LEDs, three cameras and several sensors located in different parts of its body that allow for perceiving the surrounding environment with high precision. In this work, only the information provided by the two identical RGB cameras, with a resolution of 320 × 240 pixels, situated on the forehead of the robot has been used (see Figure 3). The images of both cameras have been combined to obtain a wider field of view and better capture the person in front of the robot, thus obtaining an image of 320 × 480 resolution. An example of the combined image is shown in Figure 1a.

**Figure 4:**
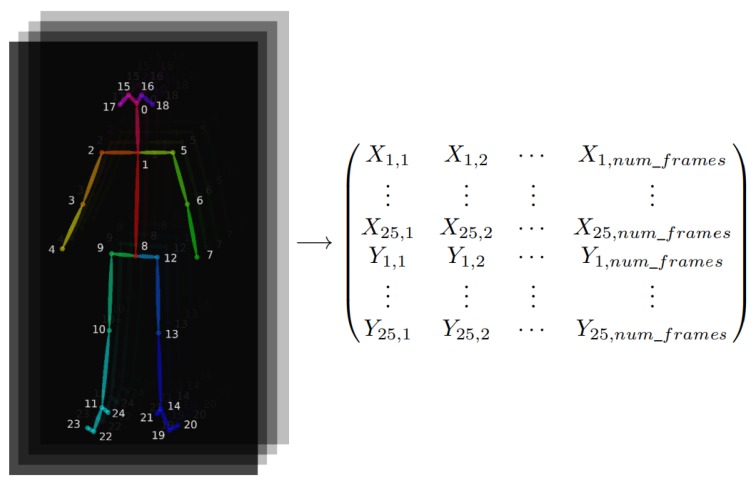
Skeleton’s joint positions and matrix representation of the extracted signals.

In order to obtain the data to apply CSP, as a first step, the skeleton of the person appearing in the scene has to be obtained. For this purpose, it has been decided to extract the skeletons using OpenPose [12], one of the most popular bottom-up approaches for multiperson human pose estimation. As with many bottom-up techniques, OpenPose first detects parts (keypoints) belonging to every person in the image and then assigns those parts to distinct individuals. The assignment is made using a nonparametric representation of association scores via Part Affinity Fields (PAFs), a set of 2d vectors fields that encode the location and orientation of limbs over the image. OpenPose can detect human body, feet, hands, and facial keypoints (135 keypoints in total) on single images. Due to the high computational cost that estimating all the keypoints requires, in this work only the BODY_25 (COCO [46] + feet) model has been used for human pose estimation. It returns the (X,Y) positions in the image of the extracted 25-keypoints, including head, body, and feet (see Figure 4).

### 4.2. Dataset

The videos in the database have been recorded using the combined image obtained from Pepper’s forehead cameras. It consists of 272 videos with six action categories and around 45 clips belong to each category, performed by 46 different people. The robot adjusts the orientation of its head according to the location of the face of the person appearing in its field of view.

All the participants in this study gave their consent in being recorded for this research purpose. No raw video data has been stored, and only minimum information about joints’ spatial coordinates has been maintained. All this data is anonymised, with no information about sex, age, race, or any other condition of the participants.

The action categories and video information can be seen in Table 1.

These are the six categories that the robot must differentiate:

1. COME: gesture for telling the robot to come to you.
2. FIVE: gesture of “high five”.
3. HANDSHAKE: gesture of handshaking with the robot.
4. HELLO: gesture for indicating hello to the robot.
5. IGNORE: ignore the robot, pass by.
6. LOOK AT: stare at the robot in front of it.

**Figure 5:**
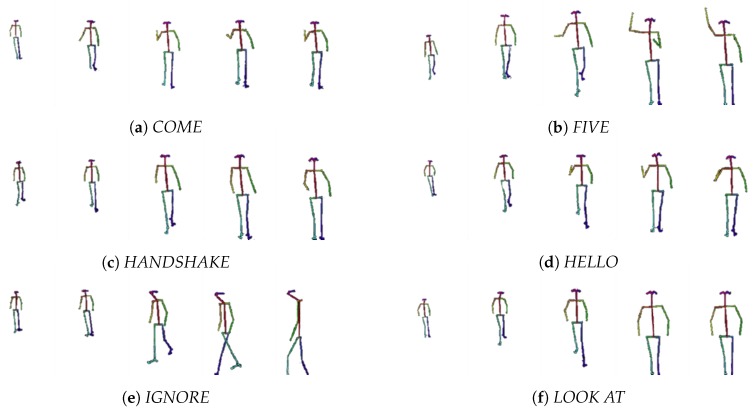
Frame sequence examples for different categories.

Examples of skeletons extracted from videos of the six different classes are shown in Figure 5. It can be seen in the examples that all the videos follow the same pattern: the actor appears in the scene, approaches the robot and finally, the action is performed.

In this case, the actions that have to be recognised are centred in the actor who performs them. Therefore, the skeleton of the actor has been extracted in every frame of each video. OpenPose returns the (X,Y) positions of 25-keypoints (joints). After obtaining the skeleton information for every frame of each video, fifty different signals are created to represent each video, where each signal will be the position of a skeleton keypoint over time. This way, there will be 50 signals (25 for the X position of the joints and another 25 for the Y position) with the same length as the original video (one skeleton per frame). The skeleton appearance and the matrix extracted from skeletons can be seen in Figure 4.

**Figure 6:**
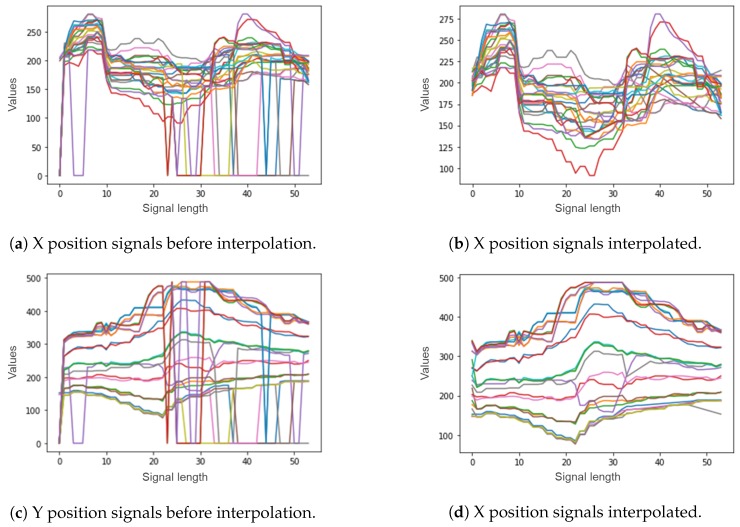
Linear interpolation example.

Some joints could be missing from the captured skeletons when the actor does not fit entirely in the camera range. In these cases, the missing joint values are estimated by a linear interpolation, using the previous and next values for that joint. The interpolation is done to avoid missing values and assuming that consecutive values of joints positions follow a smooth curve. The process of interpolation for the signal of one video can be seen graphically in Figure 6, where Figure 6a,c show the 25 X and 25 Y signals before interpolation and Figure 6b,d the 25 X and 25 Y signals after interpolating them.

Furthermore, the length of all the input data must be the same to apply the proposed method, therefore, it might be necessary to apply a preprocessing step to the videos. As the duration of the original videos differ, it has been decided to convert all the videos to the length of the longest clip.

As mentioned before, OpenPose provides the skeletons of the people of the scene for each frame of the video. It could happen that in some frames no person is detected and no skeleton is formed. Analysing this dataset, it can be noticed that full skeletons are only missed at the beginning of some of the videos and it has been decided to repeat the first skeleton encountered as many times as necessary.

After performing these changes, 50 signals with maximum video’s length are obtained. These signals are then used to feed the CSP.

### 4.3. Long Short-Term Memory (LSTM) Neural Networks

LSTMs are a category of recurrent neural networks (RNNs) which belong to the growing field of deep learning paradigms. RNNs are artificial neural networks in which connections between units form a directed cycle. Due to this architecture, recurrent neural networks possess an internal state that stores information about past inputs. This endows the recurrent networks with the ability to process sequences of inputs and exhibits a dynamic temporal behaviour in response to those sequences.

Training RNNs to learn long-term dependencies by gradient-descent methods used to be difficult due to the vanishing or exploding gradient problem [47,48]. In recent years, sophisticated optimisation techniques, specialised network designs, and new weight initialisation methods have addressed this problem with great success [49]. LSTM design introduces gates that control how much of the past and the current state has to get through to the next time step.

In a RNN, the following terms are defined:

- $x_{t}$: input vector at time step *t*.
- $h_{t}=\phi \left(Wx_{t}+Uh_{t-1}\right)$: hidden state at time step *t*. *W* and *U* are weight matrices applied to the current input and to the previous hidden state, respectively. ϕ is an activation function, typically sigmoid (σ), tanh, or ReLU.
- $o_{t}=\mathrm{softmax}\left(Vs_{t}\right)$: output vector at time step *t*. *V* is a weight matrix.

In LSTMs accounting for the capability of forgetting selectively, the node’s state is needed, so the terms are typically the following:

- $x_{t}$: input vector at time step *t*.
- $f_{t}=\sigma \left(W_{f}x_{t}+U_{f}h_{t-1}\right)$: activation vector of the forget gate at time step *t*.
- $i_{t}=\sigma \left(W_{i}x_{t}+U_{i}h_{t-1}\right)$: activation vector of the input gate at time step *t*.
- $o_{t}=\sigma \left(W_{o}x_{t}+U_{o}h_{t-1}\right)$: activation vector of the output gate at time step *t*.
- $c_{t}=f_{t}\circ c_{t-1}+i_{t}\circ \tanh\left(W_{c}x_{t}+U_{c}h_{t-1}\right)$: cell state vector at time step *t*.
- $h_{t}=o_{t}\circ \tanh\left(c_{t}\right)$: hidden state at time step *t*.

$W_{f}$, $W_{i}$, $W_{o}$, and $W_{c}$ are weight matrices applied to the current input, while $U_{f}$, $U_{i}$, $U_{o}$, and $U_{c}$ are applied to the previous hidden state. The ∘ operator represents the Hadamard product.

### 4.4. Results

Once the data have been processed, the previously explained CSP algorithm is performed. The used CSP method is implemented to work with just two classes, therefore all the tests have been carried out using pairs of classes, although multiclass classification is possible using pairwise classification approaches, such as One versus One (OVO) as a class binarization technique [50].

In Table 2 the obtained results by Linear Discriminant Analysis (LDA) classifier can be seen, and in Table 3 the results obtained by RF classifier are shown, where best results are highlighted in boldface. Both tables present the accuracy values obtained for every pair of classes of the database, using 10-fold cross validation for the evaluation. Parameter *q* indicates that only 2×q feature vectors are considered, where 2×q are the *q* first and *q* last vectors, when sorted by variance. Therefore, a feature vector of 2×q dimensionality is obtained after applying CSP, and that feature vector is the input to LDA or RF classifiers. In each table the accuracy values obtained with two different types of feature vectors are shown; variance when only the variances of the transformed signals are used to form the feature vectors and variance, max, min, IQR when apart from the variances, maximum, minimum, and IQR values are also represented in the feature vectors.

Looking at the results of Table 2, it can be observed that best outcomes are achieved when q=5, that is, taking 10 values per video is enough to perform the classification. An accuracy higher than 80% is attained for most of the category pairs. Regarding the categories, some of them are better distinguished than others. For example, good results are obtained when classifying the class ignore with all other classes, so it can be supposed that the features obtained for the category ignore are quite different from the rest. However, videos that belong to the pair of classes come and hello are more difficult to differentiate, which can be easily deduced looking at the skeletons of both classes. Concerning the feature vector type, the results indicate that there is no need to use more information than the variances of the transformed signals to obtain better results; the accuracy values obtained with the variances are higher. Nevertheless, the obtained results indicate that the presented approach yields a good classification accuracy.

The results of Table 3 show that RF classifier performs worse than LDA, obtaining lower accuracy values in general. In this case, the feature vector type which uses the variance, max, min, and IQR values achieves better outcomes. Regarding both the *q* value and the categories, the conclusions presented for the results obtained by LDA classifier are maintained.

In order to assess the effectiveness of the presented method when compared with another technique, a Long Short-Term Memory network has been chosen, as this type of neural network has been widely used for video action recognition tasks. The LSTM network has been implemented in Python using the Keras library. The input shape is bidimensional (number of frames, number of joints), and the output space is of 64 units. Then another dense layer for classification is added, of size 2, as this is the number of classes for each individual problem. The Adam optimisation algorithm [51] has been used, as well as categorical cross-entropy as loss function. It has been trained during 100 epochs, with a batch size of 25. The comparison is made between the aforementioned LSTM and the proposed approach with the configuration which has achieved highest accuracy, in this instance, variance q=5 with LDA classifier. The results are shown in Table 4, where best results are highlighted in boldface.

LSTM achieves accuracy values between 70% and 90% for most of the pairs. In this case, the accuracy obtained for come-hello pair has been improved notoriously. However, the results obtained for the rest of the classes are not that significant.

The results show that the presented method performs better than LSTM. More precisely, it outperforms LSTM results for 9 of 15 category pairs. Moreover, the mean value of all the tested pairs has been calculated for each technique, and it can be concluded that the proposed approach obtains higher accuracy values. Therefore, the CSP-based method not only achieves better results in most classifications but the average of the values obtained is higher.

Furthermore, the other three configurations tested above with LDA classifier (variance−q=10, variance−q=15 and variance, max, min, IQR−q=5) also outperform the results obtained by the LSTM method.
***variance*****q = 5**
***variance*****q = 10**
***var, max, min, IQR*****q = 5**
***variance*****q = 15**
**LSTM**0.8691>0.8622>0.8586>0.8506>0.8505

## 5. Conclusions

In this paper a new approach for activity recognition in video sequences is presented, in which Common Spatial Pattern signal processing has been applied to the skeleton joints data of people performing different activities. Features extracted from the transformed data have been used as input to Linear Discriminant Analysis and Random Forest classifiers, in order to perform action recognition. Two different sets of features have been selected: {Variance} and {Variance, Max, Min, IQR}. The results show that CSP processing followed by LDA classifier over variance features compares favourably to a Long Short-Term Memory model trained with the same data. From a database of six actions (fifteen possible pairs of actions), CSP and LDA obtains better results than LSTM in 9 of 15 category pairs.

Another advantage of the proposed method is the relative simplicity of LDA compared to LSTM networks and the lack of need for hyperparameter tuning. The set of features is also small, since only variance is used in the model that achieves best results.

As further work, it is planned to extend the range of human activities. Implementation of a real-time system could be of interest, for example, in social robotics.

## Figures and Tables

**Table 1:** Characteristics of each action category.

Category	#Video	Resolution	FPS
COME	46	320 × 480	10
FIVE	45	320× 480	10
HANDSHAKE	45	320 × 480	10
HELLO	44	320 × 480	10
IGNORE	46	320 × 480	10
LOOK AT	46	320 × 480	10

**Table 2:** Results obtained applying Common Spatial Patterns (CSP) with different *q* values and using LDA as classifier.

	Variance			Variance, Max, Min, IQR		
**Pair of Categories**	q=5	q=10	q=15	q=5	q=10	q=15
COME-FIVE	0.7579 ± 0.13	0.8124 ± 0.12	0.7667 ± 0.17	0.7578 ± 0.12	**0.8344 ± 0.14**	0.7667 ± 0.16
COME-HANDSHAKE	**0.8668 ± 0.10**	0.8019 ± 0.12	0.6910 ± 0.17	**0.8667 ± 0.13**	0.7900 ± 0.12	0.6567 ± 0.16
COME-HELLO	**0.5334 ± 0.16**	0.5000 ± 0.09	0.5000 ± 0.14	0.4778 ± 0.16	0.4444 ± 0.09	0.4778 ± 0.15
COME-IGNORE	**0.9779 ± 0.05**	0.9667 ± 0.05	0.9667 ± 0.05	0.9667 ± 0.05	0.9667 ± 0.05	0.9444 ± 0.06
COME-LOOK_AT	0.8678 ± 0.09	**0.8900 ± 0.09**	0.8789 ± 0.11	0.8678 ± 0.10	0.8356 ± 0.14	0.8033 ± 0.14
FIVE-HAND	**0.9557 ± 0.06**	0.9333 ± 0.06	0.9223 ± 0.05	0.9333 ± 0.11	0.9000 ± 0.11	0.9000 ± 0.08
FIVE-HELLO	**0.8208 ± 0.14**	0.7986 ± 0.15	0.7764 ± 0.17	0.7750 ± 0.18	0.7528 ± 0.18	0.7319 ± 0.21
FIVE-IGNORE	**0.9668 ± 0.07**	**0.9668 ± 0.07**	0.9556 ± 0.11	**0.9667 ± 0.07**	0.9556 ± 0.11	0.9556 ± 0.11
FIVE-LOOK_AT	**0.9667 ± 0.05**	0.9556 ± 0.06	0.9556 ± 0.06	0.9556 ± 0.08	0.9556 ± 0.08	0.9011 ± 0.17
HANDSHAKE-HELLO	0.7431 ± 0.19	0.7861 ± 0.14	0.8097 ± 0.10	0.7111 ± 0.24	0.7889 ± 0.21	0.8000 ± 0.10
HANDSHAKE-IGNORE	0.9889 ± 0.04	**1.0000 ± 0.00**	1.0000 ± 0.00	**1.0000 ± 0.00**	0.9889 ± 0.04	0.9889 ± 0.04
HANDSHAKE-LOOK_AT	**0.8235 ± 0.18**	0.7789 ± 0.16	0.7567 ± 0.12	0.8122 ± 0.17	0.7467 ± 0.17	0.7456 ± 0.12
HELLO-IGNORE	0.9333 ± 0.14	0.9221 ± 0.14	0.9333 ± 0.11	**0.9556 ± 0.14**	0.9444 ± 0.14	0.9444 ± 0.11
HELLO-LOOK_AT	0.8445 ± 0.11	0.8334 ± 0.12	0.8556 ± 0.14	0.8556 ± 0.09	0.8000 ± 0.10	**0.8667 ± 0.10**
IGNORE-LOOK_AT	**0.9889 ± 0.04**	**0.9889 ± 0.04**	0.9889 ± 0.04	0.9778 ± 0.05	0.9678 ± 0.05	0.9678 ± 0.05
MEAN	**0.8691**	0.8623	0.8506	0.8586	0.8448	0.8301

**Table 3:** Results obtained applying CSP with different *q* values and using RF as classifier.

	Variance			Variance, Max, Min, IQR		
**Pair of Categories**	q=5	q=10	q=15	q=5	q=10	q=15
COME-FIVE	0.6800 ± 0.29	0.6022 ± 0.24	0.5811 ± 0.19	**0.7133 ± 0.21**	0.6244 ± 0.23	0.5922 ± 0.21
COME-HANDSHAKE	0.7000 ± 0.20	0.6900 ± 0.29	0.6344 ± 0.29	**0.7556 ± 0.16**	0.6678 ± 0.32	0.6344 ± 0.32
COME-HELLO	**0.5111 ± 0.22**	0.3889 ± 0.21	0.4222 ± 0.17	0.4889 ± 0.22	0.4222 ± 0.20	0.3889 ± 0.20
COME-IGNORE	**0.9233 ± 0.12**	0.8900 ± 0.17	0.8800 ± 0.18	**0.9233 ± 0.12**	0.8911 ± 0.15	0.8578 ± 0.20
COME-LOOK_AT	**0.8133 ± 0.23**	0.7800 ± 0.20	0.7456 ± 0.25	0.8122 ± 0.23	0.8122 ± 0.24	0.7789 ± 0.24
FIVE-HANDSHAKE	**0.8889 ± 0.17**	0.7778 ± 0.15	0.6444 ± 0.17	0.8444 ± 0.17	0.7667 ± 0.12	0.6667 ± 0.17
FIVE-HELLO	**0.6264 ± 0.22**	0.5500 ± 0.22	0.5028 ± 0.23	**0.6264 ± 0.22**	0.5361 ± 0.23	0.5236 ± 0.24
FIVE-IGNORE	0.9444 ± 0.14	0.9344 ± 0.14	0.9344 ± 0.14	**0.9556 ± 0.11**	0.9456 ± 0.11	0.9233 ± 0.14
FIVE-LOOK_AT	0.9000 ± 0.19	0.8889 ± 0.21	0.8233 ± 0.23	**0.9111 ± 0.21**	0.9000 ± 0.21	0.8556 ± 0.25
HANDSHAKE-HELLO	0.6875 ± 0.18	0.5708 ± 0.14	0.6111 ± 0.20	**0.6889 ± 0.19**	0.5819 ± 0.16	0.6556 ± 0.15
HANDSHAKE-IGNORE	**0.9789 ± 0.04**	0.9578 ± 0.07	0.9133 ± 0.12	**0.9789 ± 0.04**	0.9578 ± 0.07	0.9244 ± 0.11
HANDSHAKE-LOOK_AT	0.7344 ± 0.26	**0.7556 ± 0.29**	0.6789 ± 0.29	0.7456 ± 0.26	0.7456 ± 0.28	0.6678 ± 0.25
HELLO-IGNORE	0.9000 ± 0.14	0.8889 ± 0.17	0.8667 ± 0.21	**0.9111 ± 0.15**	0.8889 ± 0.17	0.8667 ± 0.21
HELLO-LOOK_AT	0.7667 ± 0.22	0.6556 ± 0.32	0.6556 ± 0.35	**0.7889 ± 0.23**	0.7556 ± 0.29	0.7333 ± 0.28
IGNORE-LOOK_AT	0.9222 ± 0.12	**0.9333 ± 0.14**	0.9222 ± 0.14	**0.9333 ± 0.09**	0.9111 ± 0.15	**0.9333 ± 0.14**
MEAN	0.7985	0.7509	0.7211	**0.8052**	0.7605	0.7335

**Table 4:** Comparison between the proposed approach and LSTM approach.

Pair of Categories	CSP (Variance and q=5) + LDA	LSTM
COME-FIVE	0.7579 ± 0.13	**0.8628 ± 0.11**
COME-HANDSHAKE	**0.8668 ± 0.10**	0.7739 ± 0.16
COME-HELLO	0.5334 ± 0.16	**0.7336 ± 0.17**
COME-IGNORE	**0.9779 ± 0.05**	0.9575 ± 0.06
COME-LOOK_AT	**0.8678 ± 0.09**	0.7849 ± 0.10
FIVE-HANDSHAKE	**0.9557 ± 0.06**	0.8125 ± 0.14
FIVE-HELLO	0.8208 ± 0.14	**0.9125 ± 0.07**
FIVE-IGNORE	0.9668 ± 0.07	**0.9789 ± 0.04**
FIVE-LOOK_AT	**0.9667 ± 0.05**	0.8889 ± 0.11
HANDSHAKE-HELLO	**0.7431 ± 0.19**	0.7108 ± 0.21
HANDSHAKE-IGNORE	**0.9889 ± 0.04**	0.9764 ± 0.05
HANDSHAKE-LOOK_AT	0.8235 ± 0.18	**0.8350 ± 0.12**
HELLO-IGNORE	0.9333 ± 0.14	**0.9789 ± 0.04**
HELLO-LOOK_AT	**0.8445 ± 0.11**	0.5733 ± 0.18
IGNORE-LOOK_AT	**0.9889 ± 0.04**	0.9775 ± 0.05
MEAN	**0.8691**	0.8505
